# Flavonoids: biosynthesis, biological functions, and biotechnological applications

**DOI:** 10.3389/fpls.2012.00222

**Published:** 2012-09-28

**Authors:** María L. Falcone Ferreyra, Sebastián P. Rius, Paula Casati

**Affiliations:** Centro de Estudios Fotosintéticos y Bioquímicos, Universidad Nacional de RosarioRosario, Santa Fe, Argentina

**Keywords:** flavonoid, transcription factors, genetic engineering, defense, combinatorial biosynthesis

## Abstract

Flavonoids are widely distributed secondary metabolites with different metabolic functions in plants. The elucidation of the biosynthetic pathways, as well as their regulation by MYB, basic helix-loop-helix (bHLH), and WD40-type transcription factors, has allowed metabolic engineering of plants through the manipulation of the different final products with valuable applications. The present review describes the regulation of flavonoid biosynthesis, as well as the biological functions of flavonoids in plants, such as in defense against UV-B radiation and pathogen infection, nodulation, and pollen fertility. In addition, we discuss different strategies and achievements through the genetic engineering of flavonoid biosynthesis with implication in the industry and the combinatorial biosynthesis in microorganisms by the reconstruction of the pathway to obtain high amounts of specific compounds.

## Introduction

**Figure 1:**
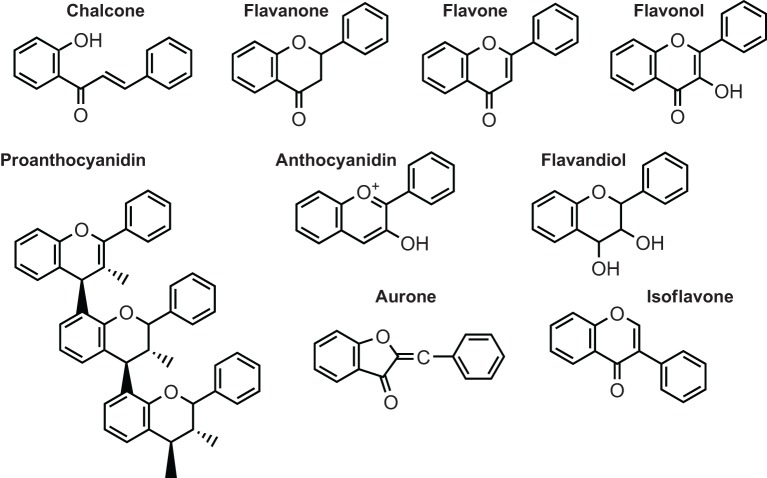
**Structure of the main classes of flavonoids**.

The pigments that color most flowers, fruits, and seeds are flavonoids. These secondary metabolites, widely distributed in plants, are classified in six major subgroups: chalcones, flavones, flavonols, flavandiols, anthocyanins, and proanthocyanidins or condensed tannins (Figure 1) and a seventh group is found in some species, the aurones (Winkel-Shirley, 2001, 2006). Legumes and a small number of nonlegume plants also synthesize specialized flavonoids such as the isoflavonoids (Yu and Mcgonigle, 2005; Miadoková, 2009; Du et al., 2010; Wang, 2011), while few species either produce 3-deoxyanthocyanins and phlobaphenes. Groups of unrelated species, including grape and peanut, synthesize stilbenes, compounds closely related to chalcones (Chong et al., 2009; Shen et al., 2009).

More than 6000 different flavonoids have been identified, and surely this number will increase (Ferrer et al., 2008). The different flavonoids have diverse biological functions, including protection against ultraviolet (UV) radiation and phytopathogens, signaling during nodulation, male fertility, auxin transport, as well as the coloration of flowers as a visual signal that attracts pollinators (Mol et al., 1998; Winkel-Shirley, 2002; Bradshaw and Schemske, 2003). Flavonoids are also responsible for the display of fall color in many plants, which may protect leaf cells from photooxidative damage, enhancing the efficiency of nutrient retrieval during senescence (Feild et al., 2001). Flavonols are probably the most important flavonoids participating in stress responses; they are the most ancient and widespread flavonoids, having a wide range of potent physiological activities (Stafford, 1991; Pollastri and Tattini, 2011).

## Flavonoid biosynthesis

Much effort has been made in elucidating the biosynthetic pathways of flavonoids from a genetic perspective. Mutants affecting flavonoid synthesis were isolated in a range of plant species. Maize (*Zea mays*), snapdragon (*Antirrhinum majus*), and petunia (*Petunia hybrida*) were established as the first major experimental models in this system, leading to the isolation of many structural and regulatory flavonoid genes (for a review, see Holton et al., 1993; Mol et al., 1998). More recently, Arabidopsis (*Arabidopsis thaliana*) has facilitated the analysis of the regulation and subcellular localization of the flavonoid pathway. An interesting aspect of using Arabidopsis for studying flavonoid biosynthesis is that single copy genes encode all enzymes of the central flavonoid metabolism, with the exception of flavonol synthase (FLS), which is encoded by six genes, but only two (FLS1 and FLS3) have demonstrated activity (Owens et al., 2008; Preuss et al., 2009). Genetic loci for both structural and regulatory genes have been identified largely based on mutations that abolish or reduce seed coat pigmentation; thus, the loci were named *transparent testa* or *tt* mutants (Koornneef, 1990; Borevitz et al., 2000). Consequently, most of the structural genes, as well as a number of regulatory genes, have been correlated with specific mutant loci in Arabidopsis. This species does not seem to use flavonoids in the same ways as some other species (for example, in defense or for male fertility); however, these mutants are helpful to define roles for these compounds in essential processes such as UV protection (Li et al., 1993; Landry et al., 1995) and the regulation of auxin transport (Murphy et al., 2000; Brown et al., 2001; Kuhn et al., 2011; Lewis et al., 2011).

**Figure 2:**
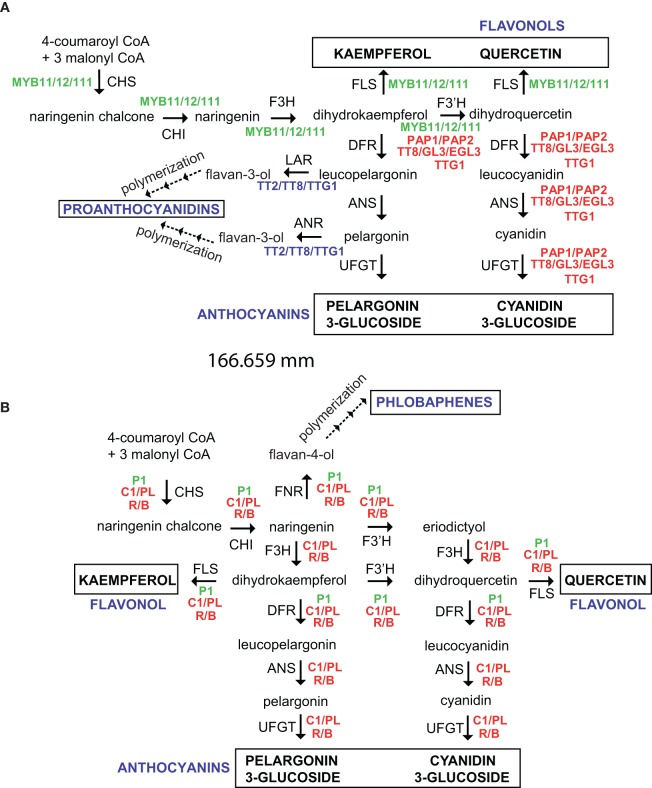
**Regulation of the flavonoid pathway in *Arabidopsis thaliana* (A) and maize (B).** Enzymes and intermediates are indicated in black and different regulators are indicated in color. End products are identified in capital letters. Dotted arrows indicate multiple steps. CHS, Chalcone synthase; CHI, chalcone isomerase; F3H, flavanone 3-hydroxylase; F3′H, flavonoid-3′-hydroxylase; DFR, dihydroflavonol 4-reductase; FNR, flavanone 4-reductase; ANS, anthocyanidin synthase; UFGT, UDP-glucose flavonoid 3-O glucosyltransferase; FLS, flavonol synthase; LAR, leucoanthocyanidin reductase; ANR, anthocyanidin reductase.

Flavonoids are synthesized through the phenylpropanoid pathway, transforming phenylalanine into 4-coumaroyl-CoA, which finally enters the flavonoid biosynthesis pathway (Figure 2). The first enzyme specific for the flavonoid pathway, chalcone synthase, produces chalcone scaffolds from which all flavonoids derive. Although the central pathway for flavonoid biosynthesis is conserved in plants, depending on the species, a group of enzymes, such as isomerases, reductases, hydroxylases, and several Fe^2+^/2-oxoglutarate-dependent dioxygenases modify the basic flavonoid skeleton, leading to the different flavonoid subclasses (Martens et al., 2010). Last, tranferases modify the flavonoid backbone with sugars, methyl groups and/or acyl moieties, modulating the physiological activity of the resulting flavonoid by altering their solubility, reactivity and interaction with cellular targets (Bowles et al., 2005; Ferrer et al., 2008).

Evidence is emerging showing that consecutive enzymes of the phenylpropanoid and flavonoid biosynthesis are organized into macromolecular complexes that can be associated with endomembranes (Kutchan, 2005). Metabolic channeling in plant secondary metabolism enables plants to effectively synthesize specific natural products and thus avoid metabolic interference. The existence of cytochrome P450 monooxygenases (P450s)-related metabolons has been demonstrated: direct and indirect experimental data describe P450 enzymes in the phenylpropanoid, flavonoid, cyanogenic glucoside, and other biosynthetic pathways (Winkel, 2004; Ralston and Yu, 2006). Additional evidence for the channeling of intermediates between specific isoforms of phenylalanine ammonia lyase and cinnamate-4-hydroxylase has been provided using transgenic tobacco plants expressing epitope-tagged versions of two phenylalanine ammonia lyase isoforms (PAL1 and PAL2) and of cinnamate-4-hydroxylase (Achnine et al., 2004). Moreover, the existence of a multienzyme complex has been proposed for the anthocyanin pathway in rice by yeast-two hybrid experiments (Shih et al., 2008).

Most of the flavonoid synthesizing enzymes are recovered in soluble cell fractions; immunolocalization experiments suggest that they are loosely bound to the endoplasmic reticulum (ER), possibly in a multi-enzyme complex, whereas the pigments themselves accumulate in the vacuole (i.e., anthocyanins and proanthocyanidins) or the cell wall (i.e., phlobaphenes Winkel-Shirley, 2001). Flavonol synthase1 has recently been localized in Arabidopsis nuclei (Kuhn et al., 2011), as well as chalcone synthase and chalcone isomerase (Saslowsky et al., 2005). Interestingly, *Antirrhinum majus* aureusidin synthase, the enzyme that catalyzes aurone biosynthesis from chalcones, was localized in the vacuole, while the chalcone 4′-*O*-glucosyltransferase is localized in the cytoplasm, indicating that chalcones 4-*O*-glucosides are transported to the vacuole and therein converted to aurone 6-*O*-glucosides (Ono et al., 2006). Moreover, a flavonoid-3′-hydroxylase has been recently localized in the tonoplast in the hilum region of the soybean immature seed coat (Toda et al., 2012).

Two models have been proposed for the mechanism of anthocyanin transport from the ER to the vacuole storage sites: the ligandin transport and the vesicular transport (Grotewold and Davis, 2008; Zhao and Dixon, 2010). The ligandin transport model is based on genetic evidence showing that glutathione transferase (GST)-like proteins are required for vacuolar sequestration of pigments in maize, petunia and Arabidopsis (AtTT19) (Marrs et al., 1995; Alfenito et al., 1998). The vacuolar sequestration of anthocyanins in maize requires a *m*ultidrug *r*esistance associated *p*rotein-type (MRP) transporter on the tonoplast membrane, which expression is co-regulated with the structural anthocyanin genes (Goodman et al., 2004). MRP proteins are often referred as glutathione S-X (GS-X) pumps because they transport a variety of glutathione conjugates. However, because anthocyanin–glutathione conjugate(s) have not been found, it is proposed that these GSTs might deliver their flavonoid substrates directly to the transporter, acting as a carrier protein or ligandin (Koes et al., 2005). This hypothesis is supported by the fact that Arabidopsis' GST (TT19), localized both in the cytoplasm and the tonoplast, can bind to glycosylated anthocyanins and aglycones but does not conjugate these compounds with glutathione (Sun et al., 2012). The vesicle-mediated transport model proposed is based on observations that anthocyanins and other flavonoids accumulate in the cytoplasm in discrete vesicle-like structures (anthocyanoplasts), and then they might be imported into the vacuole by an autophagic mechanism (Pourcel et al., 2010). Nevertheless, grape vesicle-mediated transport of anthocyanins involves a GST and two multidrug and toxic compound extrusion-type transporters (anthoMATEs). Thus, these observations point out to the coexistence of both mechanisms of transports, in which the participation of GSTs and transporters would be specific to cell and/or flavonoid-type (Gomez et al., 2011).

## Regulation of flavonoid biosynthesis

The flavonoid biosynthesis genes are regulated by the interaction of different families of transcription factors. Genes involved in the anthocyanin pathway are differentially regulated in monocot and dicot species by R2R3 MYB transcription factors, basic helix-loop-helix (bHLH), and WD40 proteins (Grotewold, 2005; Petroni and Tonelli, 2011). Thus, combinations of the R2R3-MYB, bHLH, and WD40 transcription factors and their interactions (MYB-bHLH-WD40 complex) determine the activation, and spatial and temporal expression of structural genes of anthocyanin biosynthesis. The regulation of anthocyanin biosynthesis in reproductive and other organs by MYB-bHLH-WD40 complex has been reviewed (Petroni and Tonelli, 2011). There are interesting differences in anthocyanin regulation between monocot and dicot species like Arabidopsis and maize. In Arabidopsis, TT2, TT8, and TTG1 form a ternary complex and activate proanthocyanidin biosynthesis in developing seeds, while, TTG1, a WD40 transcription factor, different bHLH (TT8, GL3, and EGL3) and MYB transcription factors (PAP1 and PAP2) interact to activate anthocyanin synthesis in vegetative tissues (Figure 2A) (Baudry et al., 2004; Feller et al., 2011). In maize, MYB and bHLH proteins are encoded by two multigene families (*PL*/*C1* and *B*/*R*, respectively), and each member has a tissue- and developmental-specific pattern, while a WD40 protein PAC1 is required by both B1 or R1 proteins for full activation of anthocyanin biosynthetic genes in seeds and roots (Figure 2B) (Carey et al., 2004). Functional Arabidopsis TTG1 is required for anthocyanin accumulation during roots and trichomes development (Galway et al., 1994), and maize PAC1 can complement Arabidopsis *ttg1* mutants; however, maize *pac1* mutants only show a reduction in anthocyanin pigmentation in specific tissues (Carey et al., 2004). Even more, the regulation of flavonol biosynthesis exhibit important differences between both species. In Arabidopsis, three R2R3-MYB proteins, MYB12, MYB11, and MYB111 (PFG1-3), which exhibit differential spatial expression patterns, regulate *AtFLS1* expression in a tissue- and developmental-specific manner (Stracke et al., 2007); while, *ZmFLS1/2* are regulated by both P1 (R2R3-MYB) and the anthocyanin C1/PL1 and R/B regulators (Figure 2) (Falcone Ferreyra et al., 2012). Nevertheless, flavonols are essential for pollen germination and conditional male fertility in maize (Mo et al., 1992; Taylor and Hepler, 1997), but maize plants lacking the P1 and R/B+C1/PL1 anthocyanin regulators are fertile (Coe and Neuffer, 1988; Dooner et al., 1991; Neuffer et al., 1997). In addition, a PFG1-3-independent flavonol accumulation occurs in pollen and siliques/seeds in Arabidopsis (Stracke et al., 2010), suggesting that, in both species, additional regulators, not yet identified, are also involved in the regulation of *FLS* expression, and consequently, in flavonol accumulation.

In addition, the evolution of MYB and bHLH plant families has been deeply analyzed from structural and functional perspectives (Feller et al., 2011). Interestingly, the identification of a C1-like (MBF1) regulator in the gymnosperm *Picea mariana* (black spruce) further supports the idea that the regulation of anthocyanin pathway by a C1-like class of R2R3 MYB protein precedes the evolutionary separation of angiosperms from gymnosperms (Xue et al., 2003). The identification of both bHLH and MYB proteins in mosses further supports the hypothesis that the bHLH–MYB complex has evolved early during land plant evolution (Pires and Dolan, 2010).

Many R2R3 MYB transcription factors were first identified from several model plants, such as maize, *Antirrhinum*, petunia, and Arabidopsis. These transcription factors are involved in the regulation of the flavonoid biosynthesis pathway. The increasing availability of plant genomes has allowed the identification and isolation of a large number of *MYB* genes involved in the regulation of flavonoid biosynthesis from diverse non-model plant species such as grapevine (*Vitis vinifera*), strawberry (*Fragaria x ananassa*), apple (*Malus domestica*), cauliflower (*Brassica oleracea var botrytis*), potato (*Solanum tuberosum* L.), bayberry (*Myrica rubra*), mangosteen (*Garcinia mangostana* L.), pear (*Pyrus pyrifolia)*, and purple kale (*Brassica oleracea* var. *acephala f. tricolor*) (Hichri et al., 2011). Furthermore, many of these regulators have been functionally characterized by transient experiments and stable expression in heterologous vegetal hosts. Table 1 shows examples of MYB transcription factors that regulate flavonoid biosynthesis.

**Table 1:** **MYB transcription factors involved in the regulation of flavonoid biosynthetic genes**.

**Species**	**MYB**	**Target genes/functions**	**References**
*Zea mays* (maize)	ZmP1	Regulation of 3-deoxyanthocyanin, phlobaphene and flavonol biosynthesis	Grotewold et al., 1998; Bruce et al., 2000; Ferreyra et al., 2010
*Sorghum bicolor*	SbY1	CHS, CHI, DFR Regulation of 3-deoxyflavonoid biosynthesis	Du et al., 2009
*Antirrhinum majus* (snapdragon)	AmMYB305	PAL, CHI, F3H	Du et al., 2009
	AmMYB340	Regulation of flavonol biosynthesis	
*Solanum tuberosum* (potato)	StD	F3H, DFR, F3′5′H	Jung et al., 2009
		Regulation of anthocyanin biosynthesis in tuber skin	
*Nicotiana tabacum*	NtAN2	Regulation of anthocyanin biosynthesis in flowers	Pattanaik et al., 2010
*Brassica oleracea* var *botrytis* (cauliflower)	Pr-D	F3H, DFR, ANS	Chiu et al., 2010
		Regulation of anthocyanin biosynthesis	
*Vitis vinifera* (grape)	VvMYBF1	Regulation of flavonol biosynthesis in developing grape berries	Czemmel et al., 2009
	VvMYBPA1	LAR1, ANR	Bogs et al., 2007
	VvMYB5a	Regulation of proanthocyanidin biosynthesis in developing grape berries	
	VvMYB5b		
	VvMYB5b	Regulation of anthocyanidin biosynthesis in ripening grape berries	Deluc et al., 2008
	VvMYBA1		
	VvMYBA2		

## Biological functions of flavonoids

A variety of derivatives of the initial phenylpropanoid scaffold serve vital roles in plant structural integrity, UV photoprotection, reproduction, and internal regulation of plant cell physiology and signaling. Phenylpropanoids also act as key chemical modulators of plant communication with insects and microbes, either as attractants or repellants, as phytoalexins against pathogens and herbivores, and as attractants to pollinators via flower color. They also induce root nodulation when excreted by symbiotic nitrogen-fixing rhizobia (Mandal et al., 2010).

The biological functions of flavonoids are linked to their potential cytotoxicity and their capacity to interact with enzymes through protein complexation. Some flavonoids provide stress protection, for example, acting as scavengers of free radicals such as reactive oxygen species (ROS), as well as chelating metals that generate ROS via the Fenton reaction (Williams et al., 2004). Flavonoids are also involved in the resistance to aluminum toxicity in maize. Roots of maize plants that were exposed to aluminum exuded high levels of phenolic compounds such as catechin and quercetin; indicating that their ability of chelating metals can be an *in vivo* mechanism to ameliorate aluminum toxicity (Kidd et al., 2001).

Evidence links flavonoids with the control of the polar transport of auxins. This hormone probably has a role in the stress response by controlling stomatal opening and by allocating resources under poor growth conditions (Peer and Murphy, 2007; Kuhn et al., 2011; Lewis et al., 2011). Flavonoids, such as quercetin, kaempferol, apigenin, and other aglycone molecules synthesized in the first steps of the flavonoid biosynthesis pathway, inhibit polar auxin transport and enhance consequent localized auxin accumulation *in planta* (Peer and Murphy, 2007; Kuhn et al., 2011; Lewis et al., 2011).

### Roles of flavonoids in legume-rhizobial interactions during nodulation

Flavonoids are involved in the nodulation process. Flavonoid-deficient roots of transgenic plants produced by RNA interference of *chalcone synthase* were unable to initiate nodules (Wasson et al., 2006). The complementation of the nodulation and flavonoid deficiency in roots by exogenous naringenin and liquiritigenin, precursors converted to the usual end product in *Medicago truncatula* and soybean, indicated that the lack of flavonoids was the reason for nodulation deficiency. In addition, these flavonoid-deficient roots had also increased auxin transport and lacked local inhibition of auxin transport at the site of nodulation (Wasson et al., 2006). However, an isoflavone-hypersensitive *Rhizobium* strain that requires very low levels of isoflavones to induce the *nod* genes is able to normally nodulate isoflavone-deficient roots, indicating that nodule primordia can form even in the absence of auxin transport inhibition by isoflavones in soybean roots. In conclusion, isoflavone-mediated auxin transport inhibition is not always essential for soybean nodulation (Subramanian et al., 2006). By silencing different *M. truncatula* flavonoid-biosynthesis enzymes, (*isoflavone synthase*, *chalcone reductase*, *flavone synthase*, and *chalcone synthase*), it was demonstrated that flavones and flavonols may act as internal inducers of rhizobial *nod* genes and auxin transport regulators during nodulation by *Sinorhibium meliloti*, respectively, (Zhang et al., 2009). In contrast to the increased auxin transport in isoflavone-null roots of soybean, isoflavone-null roots of *M. truncatula* showed unaltered auxin transport indicating that legumes use different flavonoid compounds to regulate auxin transport during nodulation (Zhang et al., 2009).

### Flavonoids in plant defense

#### Responses against UV-B radiation

The UV-absorbing characteristics of flavonoids have long been considered as evidence for the role of flavonoids in UV protection. Studies in a wide range of species, such as *Ligustrum vulgare, Vitis vinifera*, petunia, and Arabidopsis have provided new evidence that UV light induces the synthesis of flavonol compounds (Ryan et al., 2002; Berli et al., 2010; Stracke et al., 2010; Agati et al., 2011; Kusano et al., 2011). Because the presence of the OH group in the 3-position of the flavonoid skeleton is the main structural feature responsible in chelating metal ions such as iron, copper, zinc, aluminum, and hence, inhibiting the formation of free radicals as well as to reduce ROS once formed, it was suggested that flavonols might play yet uncharacterized roles in the UV stress response (Verdan et al., 2011). Furthermore, grass species such as *Deschampsia antarctica*, *Deschampsia borealis*, and *Calamagrostis epigeios* that grow in regions with elevated levels of solar UV-B radiation have high constitutive levels of flavonoids like the flavones orientin and luteolin, that protect plants against this stress condition (Van De Staaij et al., 2002). Similarly, maize plants growing at high altitudes accumulate *C*-glycosyl flavones in leaves, maysin and its biosynthetic precursor rhamnosylisoorientin, flavones commonly found in silks, as a mechanism that prevents damage caused by high UV-B exposure (Zhang et al., 2003; Casati and Walbot, 2005). *FLS* genes are regulated by UV-B radiation in both high-altitude landraces and low-altitudes inbreds of maize. Higher transcript levels are present in high-altitude plants where there are high levels of UV-B radiation than at low-altitudes. Consequently, considering the protective role of flavonols to UV-B radiation, we hypothesize that the high transcript levels of *ZmFLS* genes may also contribute to the adaptation to this stress condition with higher UV-B fluxes (Falcone Ferreyra et al., 2012).

#### Responses against infection

Flavonoids protect plants against pathogen and herbivores. According to the phytochemical co-evolution theory, the secondary metabolites are likely the most important mediators of plant-insect interactions. Thus, both plants and insect herbivores have evolved leading to the plant defense (i.e., plant secondary metabolites) and herbivore offense (i.e., detoxification ability) (Cornell and Hawkins, 2003; Kliebenstein, 2004; Bidart-Bouzat and Imeh-Nathaniel, 2008). Human-induced changes in abiotic environmental factors such as atmospheric CO_2_ and ozone (O_3_) levels, UV light, changes in precipitation patterns or temperature may directly affect the concentration of secondary chemicals in plants, which in turn may influence levels of herbivory or pathogen attack. For example, UV-B radiation modifies the production of secondary metabolites with photoprotective qualities (Mazza et al., 2000; Bassman, 2004), such as anthocyanins, isoflavonoids, and flavonol glycosides. The induction of UV-absorbing chemicals is shared with plant responses to other stresses, such as herbivore or pathogen attack, and this induction may act either positively or negatively on the levels of phytochemical production. Genes associated with the response of *Nicotiana longiflora* plants to insect herbivory were also induced by UV-B radiation (Izaguirre et al., 2003). Conversely, there is evidence that the induction of the flavonoid biosynthesis pathway by UV light can be inhibited by pathogen-induced defense responses in parsley (*Petroselinum crispum*) (Logemann and Hahlbrock, 2002). Furthermore, genes regulating the phenylpropanoid pathway leading to the synthesis of phenolic compounds like flavonoids are regulated by both UV light levels and herbivory (reviewed in Stratmann, 2003). Thus, Arabidopsis plants respond to the combination of biotic (bacterial elicitor flg22) and abiotic stresses (UV-B radiation) through the synthesis of defense-related compounds such as phytoalexins and lignin as structural barriers to restrict pathogen spread, and modify the expression of genes involved in the production of protective metabolites such as flavonols. This crosstalk involves antagonist regulation of two MYB transcription factors, the positive and negative regulators, MYB12 and MYB4, respectively (Schenke et al., 2011).

Genes expressed in incompatible and compatible plant-microbe interactions were identified by using a large-scale transcript profiling analysis of soybean and *M. truncatula.* There was a sharp and rapid up-regulation of genes encoding enzymes involved in the phenylpropanoid pathway, in particular for the synthesis of isoflavones and isoflavanones (Samac and Graham, 2007). The responses of soybean to avirulent and virulent strains of the bacterial pathogen *P. syringae* pv. *glycinea*, differing in the presence or absence of *avrB*, were investigated using a cDNA array (Zou et al., 2005). Decreased levels of transcripts specific to the anthocyanin branch of the flavonoid pathway were observed. The largest group of up-regulated genes included genes involved in the flavone and isoflavone biosynthesis. Thus, it is suggested that the opposite regulation of these branches enhances production of isoflavones that act as antioxidants and antimicrobial compounds vs. those responsible for color (Samac and Graham, 2007).

The response of susceptible soybean “Essex” and a partially resistant *r*ecombinant *i*nbred *l*ine (RIL23) to the fungal pathogen *Fusarium solani* f. sp. *glycines*, the causal agent of Sudden Death Syndrome, was studied (Iqbal et al., 2005). Up-regulation of several genes encoding enzymes in the phenylpropanoid pathway was observed in RIL23, suggesting that the products of this pathway participate in the resistance to Sudden Death Syndrome. The transcription of phenylpropanoid metabolism genes was up-regulated (along with others) after challenging susceptible soybean with the pathogen *Phytophthora sojae* (Moy et al., 2004). Isoflavones are also important in *R*-gene-mediated resistance to *P. sojae*; RNAi down-regulated *isoflavone synthase* genes in soybean roots resulted in a 95% reduction in isoflavone accumulation and an enhanced susceptibility to the pathogen (Subramanian et al., 2005).

Sustained up-regulation of genes involved in the phenylpropanoid metabolism has been associated with *R*-gene-mediated resistance responses in *M. truncatula* responding to foliar pathogens. Expression profiling of the response of two *M. truncatula* genotypes (one susceptible and one resistant) to the fungal pathogen *Colletotrichum trifolii* showed that both genotypes respond to the infection by up regulation of genes encoding phenylpropanoid pathway enzymes (Torregrosa et al., 2004). In a similar way, up-regulation of genes involved in the phenylpropanoid pathway, particularly those leading to isoflavone and isoflavonoid compounds was reported in the response of *M. truncatula* to abiotrophic pathogen, *Erysiphe pisi*, the causal agent of powdery mildew (Foster-Hartnett et al., 2007).

Corn earworm, *Helicoverpa zea*, is a major pest of maize (Ortega et al., 1980). Thus, interest in achieving maize with resistance to corn earworm has increased. One type of natural resistance is associated with the presence in silks of a *C*-glycosyl flavone: maysin, as well as related compounds: apimaysin and methoxymaysin (Waiss et al., 1979; Elliger et al., 1980; Snook et al., 1994). These compounds are insecticidal to *H. zea* larvae and are thought to interfere with the amino acid metabolism in the insect gut through their subsequent conversion to more toxic quinones. Quinones reduce the availability of free amino acids and proteins by binding to –SH and –NH_2_ groups (Byrne et al., 1997). Using flavone synthesis as a model *q*uantitative *t*rait *l*ocus (QTL) system, it was shown that in a population segregating for functional and nonfunctional *p1* alleles, the *p1* locus is the gene underlying the major QTL for maysin concentration and activity against the earworm (Byrne et al., 1996, 1997). Transgenic maize over-expressing the *p1* gene had increased silk maysin level (Johnson et al., 2007). The transgenic plants were more resistant to earworm larvae, increasing insect mortality levels and decreasing mean weights of surviving larvae. Larval weight was significantly negatively correlated with maysin level in transgenic silks.

### Flavonoids in pollen: roles in plant reproduction and fertility

The unique structure and combination of different flavonoids in each species produce yellow pollen with a range of visible and UV reflection spectra that can be detected by the targeted insects and larger animals, facilitating successful pollination. The flavonoids impart a distinctive yellow color to pollen and can be 2–4% of the dry weight (Zerback et al., 1989; Van Der Meer et al., 1992). The existence of “white pollen” has been reported in species as diverse as bristle cone pine and morning glory. The correlation between pollen fertility and flavonoids was first established in wind pollinated maize, with its numerous and well-characterized anthocyanin mutants (Mo et al., 1992). Flavonoid-deficient mutants lacking chalcone synthase were generated in maize and petunia to elucidate the roles of flavonoids in pollen (Pollak et al., 1993). These mutants were not only deficient in flavonoids but were also male sterile due to a failure to produce a functional pollen tube. This deficiency could be reversed by adding the flavonol kaempferol at pollination (Mo et al., 1992). These mutant plants are conditionally male fertile, as flavonoid-deficient pollen does not function in self-crosses but it is partially functional on wild-type stigmas containing flavonols (Mo et al., 1992).

The silencing of *chalcone synthase* gene results in parthenocarpy in tomato, but it was not identified if the cause of this phenomenon was the lack of flavonan-3-ols and/or flavonols (Schijlen et al., 2007). The silencing of a *FLS* in tobacco causes production of less-seeded fruits, and silenced lines had lower flavonol and anthocyanidins levels, while the flavan-3-ol content is increased. In addition, the pollen of these silenced lines was unable to produce functional pollen tubes. This capacity can be reversed with quercetin (*in vivo* and *in vitro*); implying that flavonols (in particular quercetin) have essential roles in pollen germination and consequently in plant fertility (Mahajan et al., 2011). Moreover, the results described indicate that the silencing of *FLS* could represent a new strategy to obtain plants with seedless/less-seeded fruits but fertility can be chemically restored for seed production.

Arabidopsis mutant plants in the *chalcone synthase* gene, which are completely devoid of flavonols in flowers and stamens, are fertile and have no pollen tube growth aberrations, indicating that flavonols are not universally essential for pollen fertility (Burbulis et al., 1996; Ylstra et al., 1996). The Arabidopsis polyketide synthases (LAP5 and LAP6) are required for pollen development and sporopollenin biosynthesis (Kim et al., 2010). Single and double mutants in *LAP5/6* have reduced to undetectable levels of different flavonoids in developing anthers and lack exine deposition causing male sterility. Both anther-specific enzymes produce the triketide and the tetraketide α-pyrones required for sporopollenin synthesis by condensation of hydroxyl fatty acyl-CoAs with malonyl-CoA. Although it was suggested that both enzymes could be involved in the synthesis of alkyl pyrones and phenolic constituents of sporopollenin in exine (Dobritsa et al., 2010), flavonoids are not produced by the action of these enzymes (Kim et al., 2010). Therefore, pollen grain cell walls defective in exine in the *lap* mutants may be deficient in the deposition of extracellular pollen coat tryphine that contains flavonoids. Consequently, the reduced flavonoid levels can be an indirect result of reduced deposition of flavonoid-containing tryphine.

## Engineering the flavonoid pathway: phenotype in mutants and over-expressing plants. Applications in industry

The engineering of the flavonoid pathway for the purposeful accumulation of compounds has been extensively used in industry (Tanaka et al., 2008). More than 20 years have passed since the color of a plant was modified for the first time through genetic engineering: the brick red petunia accumulating pelargonidin that expressed the maize *dihydroflavonol reductase* gene (Meyer et al., 1987; Nakamura et al., 2006). A huge number of genetic engineering attempts have been described to produce novel flower colors in several plant species, such as petunia, gerbera, rose, carnation, lisianthus, and torenia, by modifying the flavonoid biosynthesis pathway, either by transcriptional down-regulation, by inactivating the key enzymes of the anthocyanin pathway, or by heterologous expression of key enzymes (Tanaka et al., 2009, 2010; Nishihara and Nakatsuka, 2011). The choice of the gene source is important for an efficient change of the pathway. However, although some successful examples of color modification have been described in rose, carnation and *Nierembergia*, undesirable results were also reported probably because tissue-specific promoters were not used, adding to the difficulty in obtaining stable phenotypes (Tanaka et al., 2009, 2010). *Passiflora* species have economical importance due to the use as ornamentals, having an enormous floral diversity among species, including variation in size, morphology, fusion of floral organs, and a wide range of pigmentation patterns of the corona filaments provided by different types of anthocyanin molecules. EST databases were used to assemble sequences of *P. edulis* and *P. suberosa* corresponding to 15 different genes of the anthocyanin biosynthesis pathway as well as regulatory factors, providing useful resources for research and manipulation of secondary metabolism using transgenic approaches (Aizza and Dornelas, 2011). The results of such engineering are predictable at least to some extent. However, it is not easy to predict the amount of the compounds accumulated. In addition, the engineering of flavonoid biosynthetic pathways may affect other metabolic pathways in plants and may result in detrimental effects (Tanaka et al., 2008).

**Figure 3:**
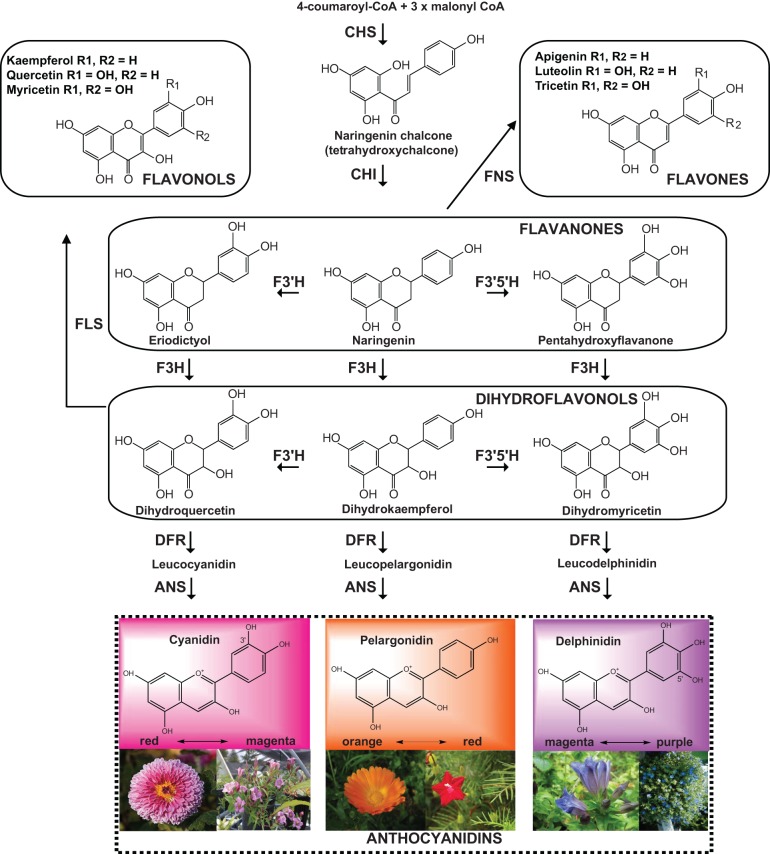
**Biosynthesis of anthocyanidins: cyanidin, pelargonidin and delphinidin.** The simplified scheme comprises the anthocyanidin branch and other flavonoid end products (flavonols and flavones). The enzymes catalyzing each step are indicated in bold. CHS, Chalcone synthase; CHI, chalcone isomerase; FNS, flavone synthase; F3H, flavonoid-3-hydroxylase; F3′H, flavonoid-3′-hydroxylase; F3′5′H, flavonoid 3′,5′-hydroxylase; FLS, flavonol synthase; DFR, dihydroflavonol 4-reductase; ANS, anthocyanidin synthase. The pictures of flowers correspond to (left to right): *Chrysanthemum morifolium*, pink gentian (*Gentiana scabra*), *Calendula officianalis*, *Ipomoea quamochit*, blue gentian (*Gentiana triflora*) and *Evolvulus pilosus*.

The three major anthocyanins pelargonidin, cyanidin, and delphinidin, contribute to orange to red, red to magenta, and magenta to purple colors, respectively (Figure 3). Methylation of cyanidin and delphinidin leads to three additional classes of anthocyanins: peonidin, petudin, and malvidin. The final color of a flower is determined by different factors that contribute to the spectrum and intensity of color: (1) the type and level of anthocyanins (determined by the anthocyanin biosynthetic pathway); (2) the complexing of anthocyanins with metal ions (producing a variety of bluish or purple hues); (3) the presence of co-pigments (flavones or flavonols); (4) the modification of basic anthocyanins (hydroxylation, methylation, acylation or conjugation); and, (5) the variation in vacuolar pH (Yu and Mcgonigle, 2005). In order to achieve a specific color by accumulating a corresponding compound, it is necessary to up-regulate the pathway leading to the compound and down-regulate the competing pathways. Sometimes, the existence of feedback inhibition in the flavonoid pathway, or certain down-regulation of a biosynthetic enzyme might destabilize a suggested metabolic biosynthetic complex (Winkel, 2004; Tanaka and Ohmiya, 2008). Consequently, engineering a pathway may have unexpected effects on a different pathway, especially if there is cross-talk between them. Different strategies have been applied to the modification of the flavonoid pathway, such as antisense, sense suppression (co-suppression), and RNAi for the down-regulation. An increase in the amount of flavonoids can be achieved by the over-expression of one of the biosynthesis or regulatory genes. Some transgenes in plants are prone to be silenced by methylation, producing variations at the transcriptional level, phenotypic differences and consequently affecting the levels of accumulation of a determined compound (Elomaa et al., 1995). Some examples of genetically modified plants in the flavonoid pathway are described below.

The substrate specificity of an enzyme can greatly contribute to determining the B-ring hydroxylation pattern. This variation influences the color of anthocyanins and, therefore, the flower/fruit color. For example, dihydroflavonol reductase from petunia efficiently utilizes dihydromyricetin but does not utilize dihydrokaempferol, and thus, this species does not accumulate pelargonidin, resulting in varieties lacking orange or bright-red flowers (Figure 3). In the case of maize and gerbera, dihydroflavonol reductase can utilize dihydrokaempferol as a substrate; thus, the generation of transgenic petunia plants expressing maize or gerbera dihydroflavonol reductase allowed the accumulation of pelargonidin, bearing brick red and orange flowers, respectively (Meyer et al., 1987). *Rosa hybrida* lacks violet to blue flower varieties due to the absence of delphinidin-based anthocyanins, usually the major constituents of purple and blue flowers, because roses do not possess flavonoid 3′, 5′-hydroxylase, a key enzyme for delphinidin biosynthesis. Expression of *F3*′*5*′*H* gene from violet (*Viola x wittrockiana*) resulted in the accumulation of a high percentage of delphinidin and a novel bluish flower color in rose petals. In addition, the endogenous dihydroflavonol reductase gene was down-regulated, and the dihydroflavonol reductase gene from *Iris hollandica* and the violet flavonoid *3*′,*5*′-*hydroxylase* gene were over-expressed in a rose cultivar, producing a dominant accumulation of delphinidin in the petals. The heritability of delphinidin accumulation was confirmed in F1 and F2 progeny by hybridization breeding of transgenic roses with seed parent non-transgenic roses (Chandler and Tanaka, 2007; Katsumoto et al., 2007).

The modification of the expression of genes that encode transcription factors regulating the flavonoid pathway is also commonly used. For example, the co-expression of the *Delila* (*Del*) and *Rosea1* (*Ros1*) cDNAs, each under the control of the fruit-specific E8 promoter, led to high levels of anthocyanin throughout the fruit tissues, which were consequently purple colored (Butelli et al., 2008). This result demonstrates that the anthocyanin biosynthetic pathway can be fully switched on in fruits if activated appropriately. Some additional examples of engineering of the flavonoid biosynthesis pathway and the phenotypes obtained are described in Table 2.

**Table 2:** **Examples of metabolic engineering of the flavonoid pathway**.

**Engineered species**	**Target gene/gene donor**	**Methodology/phenotype**	**References**
*Cyclamen persicum*	*CpF3′5′H*	Suppression by antisense.	Boase et al., 2010
		Flowers turned from purple to red/pink	
*Nicotiana tabacum* (tobacco)-Arabidopsis	*O-methyltransferase* from *S. bicolor* (SbOMT3). *Stilbene synthase* from peanut (AhSTS3)	Heterologous co-expression.	Rimando et al., 2012
		Production of resveratrol.	
		Decrease in flavonoids	
*B. napus*	*isoflavone synthase* from soybean (*GmIFS2*)	Accumulation of genistein in leaves.	Li et al., 2011
		Decreased levels of flavonols.	
*Citrus paradise* (grapefruit)	*Chalcone synthase-Chalcone isomerase*	Over-expression and suppression.	Koca et al., 2009
		Decreased level of naringin in leaves.	
*Nicotiana tabacum* (tobacco)*-Petunia hybrida*	*ZmLc* (bHLH)	Accumulation of anthocyanins in tobacco flowers and petunia leaves	Lloyd et al., 1992; Bradley et al., 1998
*Solanum lycopersicum* (tomato)	*ZmC1* (R2R3 MYB) – *ZmLc* (bHLH)	Heterologous co-expression under a fruit-specific promoter.	Bovy et al., 2002
		Increase of flavonols and flavanones in fruits.	
*Solanum lycopersicum* (tomato)-*Nicotiana tabacum* (tobacco)	*SlANT1* (R2R3-MYB)	Purple leaves	Mathews et al., 2003
*Solanum lycopersicum* (tomato)	*A. majus Del* (bHLH) and *Ros1 (MYB-related)*	Heterologous co-expression under a fruit-specific E8 promoter.	Butelli et al., 2008
		High levels of anthocyanin in fruits.	
*Nicotiana tabacum* (tobacco)	*PAP1-PAP2* from *A. thaliana*	Ectopic accumulation of anthocyanins.	Borevitz et al., 2000
		Purple plants	

Secondary metabolism is intimately linked with other aspects of plant differentiation, in which transcription factors play a key coordinating role. Recent findings illustrate the complexity of regulatory networks that control flavonoid biosynthesis in Arabidopsis and other species. They also underline the close relationship between secondary metabolism and epidermal and seed differentiation in Arabidopsis, and the central role played by conserved WD40 proteins in regulating these processes (Broun, 2005). Evidence of this connection is provided by the phenotype of Arabidopsis plants that over-express the *RED* gene, which encodes a bHLH transcription factor that regulates anthocyanin biosynthesis in maize. *RED* over-expression is sufficient to correct all defects in the *ttg1* (*transparent testa glabra1*, WD-like protein) mutant, restoring trichomes, root-hair initiation, seed-coat development, and flavonoid production (Broun, 2005).

## Combinatorial biosynthesis in microorganisms

Plant secondary metabolites are thought to be the result of sophisticated evolution. The relatively large number of secondary metabolites that are biologically active are of interest in drug discovery; and the biological activity and potency of secondary metabolites are derived largely from their complex structures. Various phenylpropanoids, including flavonoids and stilbenes, possess extraordinary antioxidant activity and estrogenic, antiviral, antibacterial, and anticancer activities. The putative health-protecting functions of flavonoids have stimulated significant research toward the elucidation of their biosynthetic networks, as well as the development of production platforms using genetically tractable hosts.

One way to produce flavonoids to be used in the pharmaceutical industry is through combinatorial biosynthesis. This technique consists of an approach in which genes from different organisms are assembled in an artificial gene cluster construct for the production of a bioactive compound. For example, *Escherichia coli* and *Saccharomyces cerevisiae* systems carrying artificial biosynthesis pathways for production of plant-specific medicinal polyketides, such as flavonoids, stilbenoids, isoflavonoids, and curcuminoids have been designed (for a review, see Katsuyama et al., 2007c; Horinouchi, 2009; Marienhagen and Bott, 2012). Starting with amino acids tyrosine and phenylalanine as substrates, these different heterologous systems yielded naringenin, resveratrol, genistein, and curcumin. Also, supplementation of *E. coli* cells with various carboxylic acids as precursors resulted in the production of previously unknown compounds; and the addition of modification enzymes to the artificial pathways led to the production of known and unknown flavonols and flavones (Miyahisa et al., 2006). These microbial systems are promising for the construction of flavonoid libraries employing enzymes of various origins as members of the artificial pathway, and for efficient use of the potential microorganism hosts. One of the advantages in assembling a biosynthesis pathway for a certain product is that replacing a single enzyme chalcone synthase or stilbene synthase, belonging to different species, gives a different product, the structure of which depends on the catalytic properties of the enzyme, the number of extensions, extender substrates employed, and mode of cyclization of the extended intermediate (Horinouchi, 2009).

The success in the fermentative production of plant specific phenylpropanoids by *E. coli* carrying an artificially assembled pathway was the first example showing that a complete plant biosynthesis pathway can be established in an heterologous microorganism for production of flavanones from the amino acid precursors, phenylalanine, and tyrosine (Hwang et al., 2003; Miyahisa et al., 2005). This was also possibly accomplished in yeast, where *p*-coumaric acid was produced by expressing *phenylalanine ammonia lyase* and *cinnamate-4-hydroxylase*. Moreover, by expressing a *4-coumarate-CoA ligase* and a *chalcone synthase*, a racemic mixture of flavanones was produced in yeast (Chemler and Koffas, 2008). The addition of *chalcone isomerase* and *chalcone reductase* along with the flavanone pathway then led to the production of both 5-deoxyflavanones and 5-hydroxyflavanones (Chemler and Koffas, 2008). Additional expression of a *flavone synthase* with the flavanone pathway produced several flavones in both yeast and *E. coli*, while expression of a flavanone 3-hydroxylase along with either a flavonol synthase or a dihydroflavonol 4-reductase with a leucocyanidin reductase yielded flavonols or catechins, respectively (Miyahisa et al., 2006; Katsuyama et al., 2007a). Moreover, 7-*O*-methyl apigenin, genkwanin, having antibacterial activity against *Vibrio cholera* and *Enterococcus faecalis* and anti-inflammatory activity, was synthesized from naringenin using *E. coli* expressing *flavone synthase I* and *flavone 7-O-methyltransferase* genes from poplar (Min et al., 2009).

Metabolic engineering has also been employed to greatly improve the efficiency of *E. coli* to produce various flavonoids by focusing on increasing intracellular pools of biosynthetic pathway cofactors. For instance, anthocyanin synthesis relies on UDP-glucose as the sugar donor. Over-expression of UDP-glucose biosynthesis genes with an anthocyanidin synthase-3-*O*-glucosyltransferase (ANS-3GT) fusion in *E. coli* yielded high anthocyanin concentrations (Yan et al., 2007). Further yield improvements were obtained by deleting competitive pathways for UDP-glucose (He et al., 2008). Nevertheless, the production in large scale of flavonoid glycosides was successfully optimized by bioconversion assays in *E. coli* expressing plant UDP-sugar: glycosyltransferases, allowing the relatively low specificity of enzymes toward the substrate acceptors to obtain a large range of glycoside products (He et al., 2008).

Other examples of combinatorial biosynthesis are the production of 5-deoxyflavanones, a natural raspberry ketone, and anthocyanin in *E. coli* (Beekwilder et al., 2007; Yan et al., 2007, 2008). The genetic design used was an artificial phenylpropanoid pathway assembling enzyme from various organisms in *E. coli*, and adding further modification enzymes. In addition, the yields were greatly increased when the two subunits of acetyl-CoA carboxylase from *Corynebacterium glutamicum* were also expressed, presumably because enhanced expression of the acetyl-CoA carboxylase greatly increases the intracellular pool of malonyl-CoA, a precursor for the synthesis (Miyahisa et al., 2005). Genistein, an isoflavone, was also synthesized by combinatorial biosynthesis; its interest resides because of its phytoestrogen activity and chemopreventive actions against cancer, osteoporosis, and cardiovascular disease (Dixon and Steele, 1999; Dixon and Ferreira, 2002). Microbial production of isoflavonoids had been difficult because isoflavone synthase, a key enzyme catalyzing the production of isoflavonoids from naringenin, is a membrane-bound cytochrome P450 enzyme that requires a specific electron transfer system. Thus, yeast or fungi were used to overcome the difficulty in expressing functionally active microsomal cytochrome P450 enzymes, which are usually difficult to express in active form in bacterial cells. Therefore, genistein was produced by the use of multiple microorganisms: first, naringenin was produced from tyrosine in the *E. coli* system, and then genistein was converted from naringenin using the yeast system (Katsuyama et al., 2007c). Moreover, a high-level production of both natural and non-natural isoflavones was later achieved in yeast fed with natural and synthetic flavanones (Chemler et al., 2010). In addition, *Streptomyces venezuelae* can be used as a heterologous host for the production of plant polyketides such as flavones and flavonols from naringenin by the introduction of *flavone synthase I* from *Petroselium crispum, flavanone 3-hydroxylase* from *Citrus sinensis* and a *flavonol synthase* gene from *Citrus unshius*, respectively (Ryeol et al., 2010).

A different example of combinatorial biosynthesis is to produce previously unknown flavonoids by precursor-directed biosynthesis. A precursor is supplied to a mutant that is blocked in the early stage of the biosynthesis of a natural product. This technique was successfully applied to the production of novel secondary metabolites by supplementation of analogs of natural precursors, which, in turn, gave novel compounds. For production of previously unknown flavanones by the system, unnatural carboxylic acids, such as fluorocinnamic acids, furyl, thienyl, pyridyl, and naphthyl acrylic acids, gave the corresponding unnatural flavanones and stilbenes (Katsuyama et al., 2007a,b).

Many enzymes of bacterial origin, as well as of plant origin, have a potential to catalyze flavonoid modification, for example, to prenylated, hydroxylated and glycosylated compounds (Winkel-Shirley, 2001; Vogel and Heilmann, 2008; Caputi et al., 2012). These modification enzymes can be readily incorporated as members in the artificial biosynthesis pathways, which should lead to the construction of larger libraries of natural and novel flavonoids, stilbenoid, and curcuminoid compounds. It was possible to modify the flavanones produced in the *E. coli* system by further introducing the *flavanone 3-hydroxylase/flavonol synthase* or *flavone synthase* genes into the biosynthetic pathway. Introduction of *flavanone 3-hydroxylase* and *flavonol synthase* genes from *Citrus* species in *E. coli* led to the production of flavonols: kaempferol from tyrosine and galangin from phenylalanine (Miyahisa et al., 2006). Similarly, introduction of a *flavone synthase I* gene from *P. crispum* in *E. coli* led to the production of flavones: apigenin from tyrosine and chrysin from phenylalanine. Finally, some of the novel flavanones synthesized were further modified by flavone synthase resulting in the production of novel flavones (Miyahisa et al., 2006), suggesting that the flavone synthase enzyme can modify a broad spectrum of flavanone substrates.

## Future challenges and remaining questions

Most of the major enzymes and genes involved in the flavonoid pathways have been characterized. However, different aspects of flavonoid biology still remain unknown. For example, the expression patterns and the activities of some of the transcription factors that regulate this branched pathway have not yet been identified. In addition, there is little evidence about the existence of protein–protein interactions that form metabolic channels that increase the efficiency of this pathway; and there is little information about the transport of flavonoids into the vacuoles. The answers to these questions will be of great importance in order to achieve efficient engineering of the flavonoid pathway in plants. In addition, the use of *A. thaliana* plants as a genetic tool clearly has helped in studying different aspects of plant secondary metabolism. Data obtained using this species will allow extrapolation to other plants of commercial and agronomic interest.

The great biodiversity of plants that arose during evolution has generated a concomitant variety of flavonoid structures known to date and many to be discovered. Further analysis of different plant species will allow the discovery of novel structures and possibly new metabolic pathways. Future studies will also contribute to the improvement of floricultural, food, pharmaceutical, and chemical industries. Moreover, evidence of beneficial functions of flavonoids in human health and the use of natural compounds for the prevention and treatment of different pathologies is continuously increasing in the world; and interest will continue to grow among researchers in the coming years.

### Conflict of interest statement

The authors declare that the research was conducted in the absence of any commercial or financial relationships that could be construed as a potential conflict of interest.
